# Cervical cancer awareness, perception, and attitude among tertiary health institution students in northeastern Nigeria

**DOI:** 10.3389/fonc.2024.1415627

**Published:** 2024-06-11

**Authors:** Zakia B. Muhammad, Uchenna S. Ezenkwa, Iragbogie A. Imoudu, Dauda A. Katagum, Iliyasu Usman, Sophia H. L. George, Matthew Schlumbrecht, Bala M. Audu

**Affiliations:** ^1^ Department of Molecular Biology and Research Laboratory, Federal Medical Centre, Azare, Bauchi State, Nigeria; ^2^ Department of Anatomic Pathology, Federal University of Health Sciences, Azare, Bauchi State, Nigeria; ^3^ Department of Paediatrics, Federal Medical Centre, Azare, Bauchi State, Nigeria; ^4^ Department of Paediatrics, Federal University of Health Sciences, Azare, Bauchi State, Nigeria; ^5^ Department of Obstetrics and Gynaecology, Federal University of Health Sciences/Federal Medical Centre, Azare, Bauchi State, Nigeria; ^6^ National Health Insurance Agency, Federal Medical Centre, Azare, Bauchi State, Nigeria; ^7^ Department of Obstetrics, Gynaecology and Reproductive Sciences, Division of Gynaecologic Oncology, Sylvester Comprehensive Cancer Centre, University of Miami Miller School of Medicine, Miami, FL, United States

**Keywords:** cervical cancer, awareness, perception, attitude, prevention, tertiary health institution students, northeastern Nigeria

## Abstract

**Background:**

The devastating scourge of cervical cancer in Africa is largely due to the absence of preventive interventions, driven by low awareness and poor perception of the disease in the continent. This work is a preliminary effort toward understanding key social drivers promoting this disease in our immediate environment with a view to mitigating it.

**Method:**

Female students of two tertiary health institutions in Azare, northeastern Nigeria, were approached to participate in this cross-sectional descriptive study. A structured self-administered questionnaire was administered to consenting participants and covered questions on their socio-demographics, awareness, perception, and attitude about/toward cervical cancer and its prevention. The responses were scrutinized for coherency and categorized into themes using summary statistics, while a chi-square test was used to determine the association between awareness of cervical cancer and participant age, marital status, religion, screening uptake, and willingness to undergo screen.

**Results:**

Awareness of cervical cancer was recorded among 174/230 (75.7%) respondents who enrolled in this study; 117 (67.2%) knew that it was preventable, but only three (1.3%) respondents had undergone screening. Among the aware participants, 91 (52.3%) and 131 (75.3%) knew that sexual intercourse and multiple sexual partners are risk factors for the disease, respectively. In contrast, knowledge of the etiology was poor; 82 (47.1%) respondents who knew it was preventable had heard about human papillomavirus (HPV), while 72 (41.4%) knew that HPV causes cervical cancer. Most (78%) of the participants expressed willingness to take a human papillomavirus vaccine or undergo screening (84.6%) if made available to them. Awareness was significantly associated with participants’ age (p = 0.022) and willingness to undergo screening (p = 0.016).

**Conclusion:**

This study revealed discordance between awareness and knowledge about cervical cancer. Educational initiatives reflective of population perception/knowledge of cervical cancer are needed to mitigate the rising incidence of this disease, especially among female healthcare providers.

## Introduction

Cervical cancer is a malignant transformation of the mucosal lining of the uterine cervix caused by persistent infection by oncogenic human papillomavirus (HPV). Globally, an estimated 604,000 new cases and 342,000 deaths were recorded worldwide in 2020, with Africa alone contributing approximately one-fifth of this burden (117,360 new cases and 76,745 deaths) in 2020 (1, 2). Preventive efforts in developed countries such as screening for premalignant changes in the cervix and vaccination against HPV have drastically reduced the incidence of this deadly disease in developed countries (3, 4). These interventions are grossly limited in Nigeria and are made worse by suspected low levels of awareness and poor knowledge about the disease among the population, especially in parts of the north.

Knowledge about a disease positively affects an individual’s ability to adopt preventative strategies and increases health-seeking behavior (5). This has been previously demonstrated among Nigerian women regarding cervical cancer (6). Studies in the northwest of Nigeria demonstrated a discordantly high level of awareness but poor knowledge about the disease (7, 8). A few studies have been reported from northeastern Nigeria describing very low awareness in some people but good knowledge in others (9, 10). Given that this zone of Nigeria is largely young with a high fertility rate, which is a risk factor for cervical cancer, it is imperative that knowledge about this disease is assessed, as this will determine their likelihood to seek appropriate care. This study therefore focused on tertiary health education institution students who, due to the nature of their studies, were hypothesized to have a high awareness, adequate knowledge, and good attitude toward the disease.

## Materials, method, and study participants

### Study design and participants

This was a cross-sectional questionnaire-based study conducted among female students of the Federal University of Health Sciences (FUHSA) and Adamu Adamu College of Nursing Sciences (AACNS), both in Azare, Bauchi State. These institutions are tertiary institutions that train students in medicine, nursing, midwifery, and other allied health sciences (FUHSA) or only nursing and midwifery (AACNS). At the time of this study, both institutions were in their first year (FUHSA) and second year (AACNS) of academic activities. Thus, the students enrolled in this study had had some but minimal exposure to clinical postings and were judged to be homogenous with regard to their knowledge of diseases generally because the first-year curriculum is uniform to a large extent.

The female population of both institutions was 711, comprising 387 from FUHSA and 324 from AACNS, all of whom were eligible to participate in the study. Using the Taro Yamane formula (11), a sample size of 256 was arrived at as suitable for the population. To increase the response rate, 300 self-administered questionnaires were distributed to consenting students by the research team over a period of 5 days. The convenience sampling method was adopted given the small size and homogeneity of the study population. To ensure equitable allocation of instruments to each site, a suitable ratio of the calculated sample size was determined using the following formula (12):


Ratio of participants = (n × N1)/N,

where n is the sample size, in this case 300; N1 is the stratum size, that is, 387 students (for FUHSA) and 324 (for AACNS); and N is the total population of eligible female students (711).

From this calculation, 163 and 137 questionnaires were allotted to FUHSA and AACNS, respectively. Female students who declined consent and male students were excluded from the study.

### Study instrument

The instrument used in this study was a self-developed, self-administered questionnaire written in English containing 35 questions divided into three thematic areas (**Supplementary Datasheet 1**). The first section contained a description of the study and consent information for the participants. It also sought participant’s demographic information such as age (≤19 years, 20–29 years, and ≥30 years), religion, educational attainment, occupation, and marital status. Educational and occupational information was added to ensure that non-students were not included in the responses or could be excluded during analysis. The next section addressed questions relating to risk factors for cervical cancer such as sexual intercourse, multiple sexual partners, and the use of barrier methods to prevent sexually transmitted infection. This section tested participants’ perceptions of these risks and also evaluated what their attitudes would be in similar circumstances. The third section dealt with the awareness, knowledge, and attitude of participants toward cervical cancer screening. Questions were asked in reference to awareness of cervical cancer, its causative agent, HPV, preventive measures, screening methods, and screening uptake. Participants were also asked about their willingness to undergo screening and vaccination services if made available to them. The instrument underwent face validation by experts in public health who recommended adjustments in the order and manner of the questions.

### Data analysis

Completed questionnaires were examined for coherence, consistency, and adequacy of responses, after which the information they contained was entered into an Excel spreadsheet and cleaned prior to analysis. The Statistical Package for Social Sciences (SPSS) software version 20 was used to analyze the data. A summary statistical tool was used to determine proportions for categorical variables and presented as frequencies and percentages under thematic sections. For questions on the Likert scale, responses with strongly agree and agree were classified as good or poor perception, while strongly disagree and disagree were combined as poor or good perception, as the situation may apply. All answers marked as undecided were treated as harmful, as it shows that the respondents may act in a negative manner when the situation presents itself. Hence, the value was added to poor perception each time. The association between awareness about cervical cancer and participants’ age, religious background, marital status, uptake of cervical screening, and willingness to undergo screen was explored using chi-square, Fisher’s exact, and likelihood ratio statistics at a two-tailed p-value of <0.05.

## Result

### Respondents’ socio-demographic characteristics

**Figure 1:**
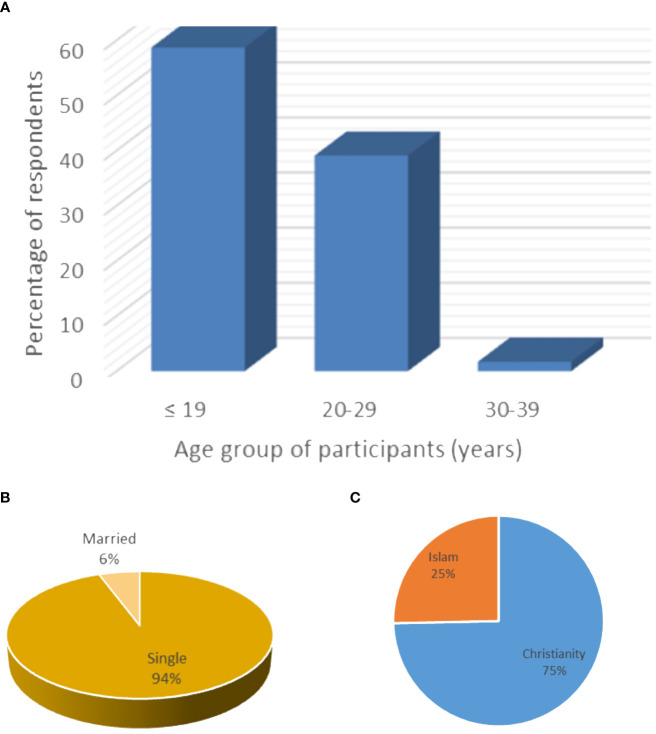
Demographic features of the respondents showing predominance of young **(A)** and single **(B)** women, most of whom identified as Christians **(C)**.

Of the 300 questionnaires distributed, 230 were completed and returned, while the remaining questionnaires were either not returned or returned uncompleted, giving a response rate of 76.7%. Among the included respondents, 45.7% (105/230) were from the AACNS, while 54.3% (125/230) were from FUHSA. Shown in **Figure 1** are the socio-demographic characteristics of the study participants. Most of the respondents, 135 (58.7%), were 19 years or younger, 217 were single (94.3%), and 172 were of a Christian religious affiliation(75%).

### Awareness/knowledge about cervical cancer

Overall, 174 (75.7%) of the respondents had heard of cervical cancer. The contents of their knowledge of the disease are shown in **Table 1**. From the table, it is readily observed that the majority of them were aware that it affects only women, and approximately three-fifths knew that the cancer is preventable if detected early. Three out of every five participants were not aware that persistent HPV infection of the cervix is the major cause of cervical cancer, and more than half of the respondents were aware that HPV vaccination can prevent cervical cancer. As few as 13.5% had heard of places where HPV vaccine can be administered.

**Table 1:** Participants’ content of awareness of cervical cancer.

Statement	Yes F (%)	No F (%)	Not sure F (%)
Cervical cancer affects only women.	149 (85.6)	22 (12.6)	3 (1.7)
Have you seen or heard of any woman affected by cervical cancer?	48 (27.6)	124 (88.5)	2 (1.5)
Cervical cancer is difficult to cure and may lead to death if not treated early.	124 (71.3)	47 (27.0)	3 (1.7)
Have you heard of human papillomavirus (HPV)?	82 (47.1)	89 (51.2)	3 (1.7)
Is cervical cancer caused by persistent HPV infection of the cervix?	72 (41.4)	99 (56.9)	3 (1.7)
Is cervical cancer preventable?	117 (67.2)	19 (11.0)	38 (21.8)

HPV, human papillomavirus.

### Cervical cancer risk factor perception

To test further the respondents’ awareness of whether cervical cancer can be prevented or not, a set of sexual behavioral risk factors were presented as Likert scale questions. Most of the respondents agreed that sexual intercourse and engaging with multiple sexual partners are risk factors for contracting cervical cancer. Approximately 10.4%–26.5% were aware that the use of barriers like condoms prevents cervical cancer. In line with the above, approximately 54% of the respondents said that they would insist on using condoms during sex even if they trusted their partners. Also, few (36.1%–34.8%) believed that protecting oneself during sexual intercourse will make a difference (**Table 2**). The summarized result for this test is presented as good or poor perception as contained in **Table 3**.

**Table 2:** Perception of the respondents about cervical cancer risk factors [F (%) = 174 (100%)].

Statement	SA F (%)	A F (%)	D F (%)	SD F (%)	UN F (%)
Sexual intercourse poses a risk of getting cervical cancer.	31 (17.8)	60 (34.5)	31 (17.8)	7 (4.0)	45 (25.9)
Multiple sexual partners increase the risk of getting cervical cancer.	59 (33.9)	72 (41.4)	13 (7.5)	3 (1.7)	27 (15.5)
Use of barriers such as condoms during sex prevents cervical cancer.	17 (9.8)	46 (26.4)	47 (27.0)	10 (5.7)	54 (31.0)
I trust my partner; therefore, I do not use condoms during sex.	13 (7.5)	22 (12.6)	50 (28.7)	25 (14.4)	64 (36.8)
I trust my partner; therefore, I do not insist on condom use during sex.	10 (5.7)	32 (18.4)	48 (27.4)	25 (14.4)	59 (33.9)
I am already sexually active; protecting myself will not make any difference.	3 (1.7)	6 (3.4)	58 (33.3)	61 (35.1)	46 (26.4)

SA, strongly agree; SD, strongly disagree; A, agree; D, disagree; UN, undecided.

**Table 3:** Perception of the respondents as good or poor regarding sexual behavioral risk factors for cervical cancer.

Question item	Good perception F (%)	Poor perception F (%)
Sexual intercourse poses a risk of getting cervical cancer.	91 (52.3)	83 (47.7)
Multiple sexual partners increase the risk of getting cervical cancer.	131 (75.3)	43 (24.7)
Use of barriers such as condoms during sex prevents cervical cancer,	64 (36.8)	110 (63.2)
I trust my partner; therefore, I do not use condoms during sex.	75 (43.1)	99 (56.9)
I trust my partner; therefore, I do not insist on condom use during sex.	73 (42)	101 (58)
I am already sexually active; protecting myself will not make any difference.	67 (38.5)	107 (61.5)

### Attitude of respondents toward cervical cancer screening and HPV vaccination

**Table 4** contains the attitude participants opined that they would adopt regarding their knowledge or awareness about the disease. Of those who had heard of cervical cancer screening (58/230; 25.2%), three persons (accounting for 1.3% of overall study participants and 5.2% of those who had heard about screening) had undergone cervical cancer screening themselves. Attitude toward screening tests was positive, as approximately 80% demonstrated a willingness to undergo cervical screening. Also, a good attitude was displayed toward HPV vaccination, with 74.8% of the respondents showing a willingness to take an HPV vaccine if made available. Less than half (34.3%) of the respondents had heard of cervical cancer screening tests, and approximately one-fifth (21.3%) knew cervical screening test centers.

**Table 4:** Knowledge about cervical cancer prevention among individuals who knew it can be prevented.

Variable	Yes	No	Unsure
Can vaccination against HPV infection prevent cervical cancer?	65 (55.6)	50 (42.7)	2 (1.7)
Have you heard of places where HPV vaccine is administered?	21 (17.9)	95 (81.2)	1 (0.9)
Would you take HPV vaccine if it is made available to you?	92 (78.6)	7 (6.0)	18 (15.4)
Have you heard of cervical cancer screening tests?	58 (49.6)	55 (47.0)	4 (3.4)
Have you heard of centers that do cervical cancer screening tests?	33 (28.2)	84 (71.8)	0 (0.0)
Have you undergone cervical screening tests in the past?	3 (2.6)	114 (97.4)	0 (0.0)
Would you like to undergo cervical cancer screening test if services are made available to you?	99 (84.6)	13 (11.1)	5 (4.3)

HPV, human papillomavirus.

### Reasons for not taking a cervical cancer screening test

The reasons for not undergoing cervical screening tests among respondents who were aware of cervical cancer, knew it is preventable, and had heard about screening but had not undergone screening are shown in **Table 5**. The most common deterrent from screening was participants’ perception that they had not met the criteria for screening (29.1%), followed by far distance from testing centers (16.3%). **Table 6** shows associations between respondents’ reports of awareness about cervical cancer and their age, religious background, marital status, uptake of screening, and willingness to undergo testing. Of these, older age was associated with being aware of the disease condition, while awareness in turn was associated with willingness to take up screening services. Religious inclination and marital status did not significantly influence awareness or willingness to undergo testing. However, all the married women who responded to the question on willingness to undergo testing if made available to them responded in the affirmative.

**Table 5:** The reasons respondents have not undergone cervical cancer screening tests.

Variable	Frequency	Percentage (%)
I don’t have cervical cancer.	5	9.1
I have not met the criteria.	16	29.1
Afraid of results.	3	5.5
Cervical cancer is not my portion.	3	5.5
My husband is not supportive.	1	1.8
It is costly.	2	3.6
It is far from where I live.	9	16.3
It is for married older women.	1	1.8
There is no time.	4	7.3
No response	11	20.0
Total	55	100.0

**Table 6:** Association between awareness and age, religious background, marital status, test uptake, and willingness to undergo screen.

Data item	Category	Awareness			Sig
		No	Not sure	Yes	
Age (years)	≤19 20–29 ≥30	34 9 0	7 4 0	90 73 4	0.022*
Religious background	Christianity Hindu Islam	16 0 27	5 0 6	36 1 130	0.123
Marital status	Married Single	0 43	0 11	12 155	0.051
Screening uptake	No Yes	40 0	10 1	161 3	0.143
Willingness to test	No Not sure Yes	7 4 26	1 1 11	4 17 162	0.016*

* Represents statistically significant p value (p < 0.05).

## Discussion

This study showed that approximately three-fourths of the respondents had heard about cervical cancer, a figure we regarded as high, considering that community-based studies in Nigeria had mostly documented a very low proportion of women who had heard about the disease. Wright et al. and Oluwole et al., both in Lagos, a cosmopolitan city, reported a disturbingly low awareness level of 37.2% and 15%, respectively (13, 14). However, a higher proportion of participants in the studies of Oluwole et al. and Wright et al. had low or no formal education, different from our population that is homogenously beyond secondary education and in health-related disciplines. This could therefore explain the higher level of awareness recorded in this study and suggests that education could have a positive effect on awareness about cervical cancer, as shown by similar previous studies among students of tertiary institutions in Nigeria (15–18). Maitanmi et al. at Babcock University Ogun State, Gborieneomie and Ibe at the University of Port Harcourt, Ogwunga et al. at the Federal University of Technology Owerri, and Ella et al. at the University of Calabar all found awareness levels of 68.4% to 98.6% (15–18). Our data are also similar to those among students in Ethiopia in the studies of Mengesha et al. and Zhang in China, with 65.1% and 87.9% awareness status, respectively (19, 20).

All the studies among students had similar age demographics and were predominantly younger than age 25 compared to the community population-based studies where more of the participants were likely to be older, as was the case in the study by Wright et al. (13). A possible unifying factor among the students therefore could be their curiosity about their sexuality and reproductive health around this age, which could prompt them to seek related knowledge. This period could profitably be exploited to pass on the right information about cervical cancer that could lead to its reduction or elimination in the near future. Our data support this position given that younger women were significantly more likely to be unaware of this health condition and its risk factors.

Low perception of risk, etiologic, and preventive factors of cervical cancer in this study was divergent from the high number of people who had been told about the disease, and so was the finding in nearly all other tertiary institutions’ reports both within and outside the country, except for that among nursing students in the University of Calabar Nigeria where a good level of knowledge was demonstrated (15–20). The majority did not recognize sexual intercourse, HPV infection, vaccination against HPV, and screening for premalignant lesions as risk, cause, and preventive factors in uterine cervix carcinogenesis. Perhaps a reasonable explanation for the positive knowledge among University of Calabar students was their advanced exposure to health information, as they were in the 300 level (year 3) of study and above in the school and could have encountered cases of cervical cancer during their rotations in the wards and theater, compared to the index study where the students of both institutions were in their first year of study with no significant exposure to such patients. There is therefore a very high need to educate our girls and women more on issues relating to their health and not to assume that being in university would address this knowledge gap.

Cervical cancer screening uptake is negligible in Nigeria, and where screening services are available, patronage is often opportunistic. Studies that have examined this occurrence have reported approximately 5% uptake in the populations studied, even among students, although one study reported a value of 14.6% among students in Calabar (18, 21). This is higher than the 2.2% screening rate seen in the present study. Reasons for this poor outcome included poor accessibility to screening centers, while some believed screening is for those already suffering from the disease. Nevertheless, other reasons were fear of the screening outcome, lack of time, cost and lack of interest, not being sexually active, husband not supportive, and belief that single and young women are not included among the at-risk individuals. Similar opinions were observed in a study from Calabar, South-Southern Nigeria (18). Fortunately, a strong willingness by the respondents to receive HPV vaccination or undergo any of the screening tests if made available to them is encouraging. This finding has also been documented by other studies in Nigeria and shows a window of opportunity for targeted interventions, especially mass health education and enlightenment, which we found can significantly influence the uptake of screening (15, 22). While this manuscript was being prepared, the Nigerian government rolled out a comprehensive HPV vaccination program, signaling a commitment to address this need in the country. Public enlightenment is now required to drive uptake and tilt over the dissenting members of the population so that there will be universal coverage. Barriers to screening such as stigmatization and embarrassment (about seeking out screening) as well as breaks in vaccine supply due to high cost should also be removed through concerted efforts by both the government and donor/non-governmental organizations to ensure sustainability (17, 23).

The results presented in this study have inherent shortcomings because they are derived from self-reports. Thus, the discordance between the high level of awareness about cervical cancer and poor perceptions about risk, causative, and preventive factors could be dependent on this. In other words, awareness level may be lower than has been reported here. What this means is that information dissemination could be laden with false content that could be as harmful as the absence of it. Second, awareness of the role that infection by the human immunodeficiency virus plays in promoting HPV infection persistence and cervical cancer progression was not assessed in this survey. Knowledge of this could prompt a higher willingness to adopt risk-reducing behaviors and also attend screening for both infections. A more controlled study is required to further examine barriers to cervical cancer control programs among the populace.

A strength of this study is the heterogeneity of the studied population comprising students in various health disciplines who were in their early years of study and whose knowledge about diseases we suspect may not differ remarkably from that of the general population. In contrast, the homogeneity of their educational background poses a limitation, making it difficult to assess the likely effect of the level of education on awareness and perception of cervical cancer. However, given that discordance between awareness and perception was documented here even among these post-secondary education level young adults, there is an urgent need to initiate a massive health campaign to disseminate the right information about the disease in order to forestall risk propagation among at-risk individuals. Notwithstanding, a cross-sectional community survey is needed to test the level of awareness about this disease.

## Conclusion

Awareness of cervical cancer and perception about its risk factors, cause, and preventive factors are divergent among the studied population and portend a significant setback in the fight against this disease, as these young women in their early reproductive age are not likely to adopt safe practices that will prevent them from developing cervical cancer in the future. Dedicated efforts should be committed to providing reliable fact-based information to the entire population. Vaccines should be made available, and parents should be encouraged to allow their children to receive them, as this is the most effective preventive method at present.

## Data availability statement

The original contributions presented in the study are included in the article/**Supplementary Material**. Further inquiries can be directed to the corresponding author.

## Ethics statement

The studies involving humans were approved by the Research, ethics and review committee, Federal Medical Centre Azare. The studies were conducted in accordance with the local legislation and institutional requirements. The participants provided their written informed consent to participate in this study.

## Author contributions

ZM: Conceptualization, Data curation, Writing – original draft, Writing – review & editing. UE: Conceptualization, Data curation, Formal analysis, Methodology, Writing – original draft, Writing – review & editing. II: Conceptualization, Methodology, Writing – review & editing. DK: Conceptualization, Methodology, Supervision, Writing – review & editing. IU: Conceptualization, Data curation, Writing – original draft. SG: Conceptualization, Methodology, Supervision, Writing – review & editing. MS: Conceptualization, Methodology, Supervision, Writing – review & editing. BA: Conceptualization, Methodology, Supervision, Writing – review & editing.
