# Supplementary material for: Cervical cancer awareness, perception, and attitude among tertiary health institution students in northeastern Nigeria
Source: Front Oncol. 2024 Jun 11;14:1415627. doi: 10.3389/fonc.2024.1415627 (PMC11196799; doi:10.3389/fonc.2024.1415627)
Supplement: Supplementary file 1 [file DataSheet_1.docx]

Questionnaire administered to the participants

**Questionnaire**

**Title of study: Cervical cancer awareness, perception and attitude among tertiary health institutions students in northeast Nigeria**

**Socio demographic Characteristics**

Please read carefully and fill in or tick (√) the correct answers in the spaces provided.

1. How old are you: less than 19 ( ), 20–29 ( ), 30–39 ( ), 40–49 ( ), 50–59 ( ), 60 and above ( ) years

2. What is your religion: Christian ( ), Moslem ( ), others ( ), specify ________________

3. What is your level of education: none ( ), primary school ( ), secondary school ( ), post-secondary ( ), others ( ),

Specify ______________

4. What is your marital status: single ( ), married ( ), divorced or separated ( ), widowed ( )

5. What is your occupation: ___________________________________________

**Perception about cervical cancer risk**

6. Sexual intercourse is a risk to getting cervical cancer:

( ) Strongly Agree ( ) Agree ( ) Undecided ( ) Disagree ( ) Strongly disagree

7. Sexual relationship with more than one partner increases the risk of getting cervical cancer:

( ) Strongly Agree ( ) Agree ( ) Undecided ( ) Disagree ( ) Strongly disagree

8. Use of barrier such as condom during sex prevents cervical cancer:

( ) Strongly Agree ( ) Agree ( ) Undecided ( ) Disagree ( ) Strongly disagree

9. I trust my partner; therefore, I do not use condom during sex.

( ) Strongly Agree ( ) Agree ( ) Undecided ( ) Disagree ( ) Strongly disagree

10. I trust my partner; therefore, I do not insist on condom use during sex.

( ) Strongly Agree ( ) Agree ( ) Undecided ( ) Disagree ( ) Strongly disagree

11. I am already sexually active; protecting myself will not make any difference:

( ) Strongly Agree ( ) Agree ( ) Undecided ( ) Disagree ( ) strongly disagree

12. I have had worrisome vaginal discharge(s) that required treatment in the past, but I do not think it could be related to cervical cancer:

( ) Strongly Agree ( ) Agree ( ) Undecided ( ) Disagree ( ) Strongly disagree

**Awareness, knowledge and attitude about cervical cancer**

13. Have you heard of cancer of the cervix: yes ( ), no ( ), not sure ( )

14. Are you aware that it affects only women: yes, I’m aware ( ), I’m not aware it affects only women ( )

15. Are you aware that it is difficult to cure and may lead to death if not treated early: yes I am aware ( ), no I am not aware( )

16. Are you aware that cervical cancer is preventable: yes ( ), no ( ), not sure ( )

17. If yes, what means can be used to prevent it? Choose all that apply: cervical cancer screening ( ), vaccination against Human papilloma virus infection ( ), safer sex ( ) I don’t know ( )

18. Have you heard of cervical cancer screening tests: yes ( ), no ( ), not sure ( )

19. Which of the cervical cancer screening tests have you heard of: Pap’s smear ( ), visual inspection with acetic acid (VIA) ( ), visual inspection with Lugol’s iodine (VILI) ( ), others ( ) (specify)_______________________________

20. Have you heard of center(s) that do cervical screening tests: yes ( ), no ( )

21. If yes, how far is(are) the center(s) from where you live: near ( ), not so near ( ), far ( ), very far ( )

22. Have you done cervical screening test in the past: yes ( ), no ( )

23. If yes, how many times: _____________________

24. What was(ere) the result(s): dysplasia ( ), cancerous, inflammatory ( ), inconclusive ( ), negative ( )

25. If no to 25 above, why have you not done cervical screening test: it is far from me ( ), it is costly ( ), there is no time ( ), it is for married women ( ), husband not in support ( ), afraid of the result ( ) I don’t have cervical cancer ( ) cervical cancer is not my portion ( ) it is for older women ( ) I have not met the criteria ( )

26. Will you like to do cervical cancer screening test if the services are made available to you: yes ( ), no ( ), not sure( )

27. Have you heard of Human Papilloma virus (HPV)? Yes ( ), No ( )

28. Do you know that cervical cancer is caused by persistent HPV infection of the cervix? Yes ( ), No ( )

29. Do you know that vaccination against HPV infection can prevent cervical cancer? Yes ( ), No ( )

30. Have you heard of places where HPV vaccine can be administered? Yes ( ) No ( )

31. Would you take HPV vaccine if it is made available to you? Yes ( ) No ( ) Not sure ( )

32. If no, why? Please briefly explain
